# Identification and characterization of skin color microRNAs in Koi carp (*Cyprinus carpio* L*.*) by Illumina sequencing

**DOI:** 10.1186/s12864-018-5189-5

**Published:** 2018-10-29

**Authors:** Mingkun Luo, Lanmei Wang, Wenbin Zhu, Jianjun Fu, Feibiao Song, Min Fang, Juanjuan Dong, Zaijie Dong

**Affiliations:** 1 Wuxi Fisheries College, Nanjing Agricultural University, Jiangsu Wuxi, 214081 People’s Republic of China; 2 Freshwater Fisheries Research Centre of Chinese Academy of Fishery Sciences, Key Laboratory of Freshwater Fisheries and Germplasm Resources Utilization, Ministry of Agriculture, Jiangsu Wuxi, 214081 People’s Republic of China

**Keywords:** miRNAs, Skin color, Characterization, Koi carp, Illumina sequencing

## Abstract

**Background:**

MicroRNAs (miRNAs) are endogenous, small (21–25 nucleotide), non-coding RNAs that play important roles in numerous biological processes. Koi carp exhibit diverse color patterns, making it an ideal subject for studying the genetics of pigmentation. However, the influence of miRNAs on skin color regulation and variation in Koi carp is poorly understood.

**Results:**

Herein, we performed small RNA (sRNA) analysis of the three main skin colors in Koi carp by Illumina sequencing. The results revealed 330, 397, and 335 conserved miRNAs (belonging to 81 families) and 340, 353, and 351 candidate miRNAs in black, red, and white libraries, respectively. A total of 164 differentially expressed miRNAs (DEMs) and 14 overlapping DEMs were identified, including *miR-196a*, *miR-125b*, *miR-202*, *miR-205-5p*, *miR-200b*, and etc. Target prediction and functional analysis of color-related miRNAs such as *miR-200b*, *miR-206*, and *miR-196a* highlighted putative target genes, including *Mitf*, *Mc1r*, *Foxd3*, and *Sox10* that are potentially related to pigmentation. Determination of reference miRNAs for relative quantification showed that *let-7a* was the most abundant single reference gene, and *let-7a* and *miR-26b* was the most abundant combination.

**Conclusions:**

The findings provide novel insight into the molecular mechanisms determining skin color differentiation in Koi carp, and serve as a valuable reference for future studies on tissue-specific miRNA abundance in Koi carp.

**Electronic supplementary material:**

The online version of this article (10.1186/s12864-018-5189-5) contains supplementary material, which is available to authorized users.

## Background

Koi carp, a colorful variant of common carp (*Cyprinus carpio* L.), has been artificially selected for centuries, and is a popular pet and hobby species that exhibits a wide variety of colors and phenotypes [1]. Unlike the slow rate of phenotypic changes occurring in wild populations, the rapid establishment of diverse colors and coloration patterns under artificial selection makes Koi carp an ideal subject for studying the genetics of pigmentation [2]. Determination of skin color is a complicated process in fish, linked to various cellular, genetic, nutritional, physiological, and environmental factors [3]. Exactly how skin color patterns form is a long standing question among biologists. Extensive studies have identified a series of genes involved in the determination of skin pigmentation, including Melanocortin receptor 1 (*Mc1r*), Microphthalmia-associated transcription factor (*Mitf*), Tyrosinase (*Tyr*), Tyrosine related protein-1 (*Tyrp1*), and Melanocyte-stimulating hormone (*Msh*) [4–6]. Additional genes related to the determination of skin color in fish remain to be elucidated.

**Fig. 1:**
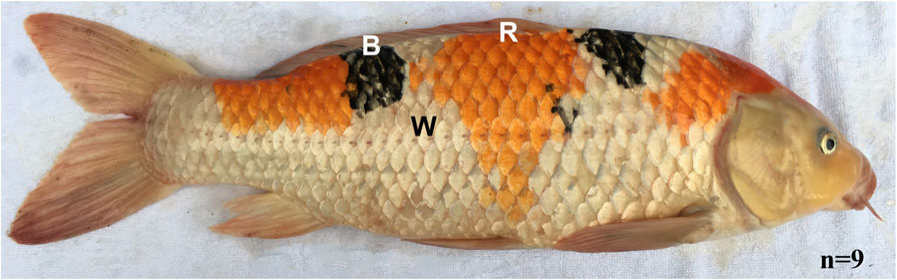
Photograph showing the three different skin color types in Koi carp. R, red; B, black; W, white; n, sample number

MicroRNAs (miRNAs) are single-stranded, non-coding, highly-conserved 19–24 nucleotides (nt) molecules that regulate gene expression at the post-transcriptional level by directly targeting RNA-inducing silencing complex (RISC) to cognate messenger RNA targets [7, 8]. Crosstalk between miRNAs and mRNAs is important for transcriptional and signal transduction events involved in multiple biological processes such as apoptosis, cell proliferation, cancer, embryo development, and skin pigmentation [9–12]. Therefore, skin-expressed miRNAs might play a vital role in skin differentiation, color formation and skin diseases. Previous studies have investigated the expression and functions of some miRNAs in animal skin. For example, the skin-specific *miR-203* was found to define a molecular boundary between proliferative basal progenitors and terminally differentiating suprabasal cells, ensuring proper identify of neighboring layers [13]. In *Drosophila*, dorsal abdomen pigmentation is decreased when *miR-8* lost [14], and coat color in mice is affected when *miR-137* is over-expressed [15]. In white alpaca (WA) and brown alpaca (BA) skin, deep sequencing identified 35 and 13 conserved differentially expressed miRNAs (DEMs), indicating potential functions in coat skin color regulation [16]. Meanwhile, Yan et al. identified 13 DEMs between red and white skin in common carp, *miR-429* is a potential regulator because it’s silencing resulted in an obvious change in skin pigmentation [12]. Thus, miRNAs could be involved in the regulation of skin color. However, the molecular and cellular mechanisms regulating skin color variation in fish, especially a variety of colors appeared on a single fish, such as Koi carp (Fig. 1), remain poorly understood.

Reference genes are employed as internal controls for normalizing real-time quantitative reverse transcription PCR (qRT-PCR) data during analysis of target gene expression, and this approach can capture all non-biological variation [17]. At present, *β-actin*, *18S rRNA*, *5S rRNA* and glycer-aldehyde-3-phosphate dehydrogenase (*GAPDH*) are most widely used as candidate reference genes for mRNA expression [18]. In typical miRNA expression studies, *18S rRNA* and small nuclear RNAs such as *U6 snRNA* are commonly used as reference genes [19, 20]. The selection of appropriate reference genes is not trivial, and studies indicate that a single gene is unlikely to behave consistently in all tissues and/or in all physiological and pathological situations [21, 22]. Therefore, internal genes should be verified and selected prudently. However, to date, few reports have analyzed the expression stability of miRNAs used as reference in teleosts.

Herein, we investigated the potential functions of miRNAs in post-transcriptional regulation of pigmentation by constructing Koi carp miRNA libraries from white, red, and black fish skin, identified hundreds of conserved miRNAs and candidate novel miRNAs, and revealed evidence of differential expression of miRNAs in different colors. We examined candidate reference miRNAs in 15 tissues (blood, brain, muscle, liver, kidney, spleen, intestine, heart, gill, fin, eye, ovary, and white/red/black skin) to minimize the risk of co-regulation artifacts. The results provide fundamental knowledge for unraveling the genetic mechanisms underlying color trait variation, and could assist the exploitation of gene regulation linked to skin color determination.

## Materials and methods

### Sample collection

The Koi carp used in this study were obtained from the aquaculture base of Freshwater Fisheries Research Center (FFRC) affiliated with the Chinese Academy of Fishery Sciences. Animals (average weight: 1000 ± 80 g) were raised at 24 ± 1 °C in 256 L tanks in a circulation water system for 1 week before experiments and fed twice daily (in the morning and late afternoon). Aeration was supplied constantly and a 12 h light / 12 h dark photoperiod was employed.

All of the surviving fish were tranquilized in 20–30 mg/L MS-222 buffered to pH 7.0–7.5, and three sets of skin tissues were collected from nine Koi carp (black, white and red spots in one fish synchronous); three black skin (BS), white skin (WS), and red skin (RS) samples per group, and another 12 tissue samples (blood, brain, muscle, liver, kidney, spleen, intestine, heart, gill, fin, eye, and ovary) were also collected. All fresh tissue samples were immediately snap-frozen in liquid nitrogen and stored at − 80 °C until use.

### RNA isolation, library preparation and sequencing

Total RNA was obtained from Koi carp samples using TRIzol (Invitrogen, UK) according to the manufacturer’s protocol, and genomic DNA was removed using DNase I (New England Biolabs). RNA purity was assessed using the Nanodrop-2000 instrument (Thermo Scientific, USA), and all RNA samples had an A260:A280 ratio > 1.9. An equal amount of total RNA from three fish individuals from each color group (black, white, and red spots) was pooled, and a total of nine RNA pools were prepared and used for library construction. A pair of Illunima adapters was ligated to the RNA 5′ and 3′ termini of RNAs, and the resultant products were reverse-transcribed and amplified through 15 PCR cycles. Fragments ranging in size from 140 to 150 nt were retained and purified to generate the sequencing libraries. Each library was loaded into a single Illunima Hiseq2500 lane for 1× 50 base pair (bp) single-end (SE) sequencing. All raw transcriptome data have been deposited in the NCBI Short Read Archive (SRA) with the accession numbers SRR7476960, SRR7476961, SRR7476962, SRR7476963, SRR7476964, SRR7476965, SRR7476966, SRR7476967, and SRR7476968.

### Analysis of sequencing data and miRNA identification

FasQC (http://www.bioinformatics.babraham.ac.uk/projects/fastqc/) was used to control the data quality. An initial filtering step was performed to exclude poor quality reads, 3′ and 5′ adaptor reads, and reads shorter than 18 nt. The remaining sequences were mapped to the *C. carpio* genome using the SOAP program (http://soap.genomics.org.cn) with a tolerance of one mismatch. Sequences matched to the *C. carpio* genome were subsequently analyzed to filter out rRNAs, tRNAs, snRNAs, snoRNAs, and non-coding RNA reads by BLAST searching against the Rfam (11.0, http://rfam.xfam.org/) and GenBank (https://www.ncbi.nlm.nih.gov/) databases. To ensure each unique small RNA (sRNA) was mapped to only one annotation, we followed the rule rRNAetc (Genbank> Rfam) > known miRNA >repeat >exon >intron3, as previously described [23]. Subsequently, the remaining reads were identified as conserved miRNAs by BLAST searching against miRBase 21.0, allowing no more than two mismatches [24]. Sequences that did not match existing or conserved miRNAs were used to identify potentially novel miRNA candidates [25, 26]. The enrichment degree of each miRNA was identified by counting the number of reads in each sample, and miRNAs were family clustered in each library through miRBase 21.0 database.

### Differential expression of miRNAs

To compare the expression levels of miRNAs among the black, white, and red skin libraries, the frequency of miRNA counts was normalized as transcripts per million (TPM). Calculated as follows: normalized expression (TPM) = (actual miRNA count/number of total clean reads) × 1,000,000. Only miRNAs with altered by more than two-fold relative to controls were considered significantly differentially expressed (*p*-value < 0.05) [27] and classified as DEMs. A positive value indicated up-regulation, while a negative value indicated down-regulation.

Fold-change was calculated as follows: Fold-change = log2 (treatment/control).

The *p*-value was calculated as follows:

$$ p\left(x|y\right)={\left(\frac{N_2}{N_1}\right)}^y\frac{\left(x+y\right)!}{x!y!{\left(1+\frac{N_2}{N_1}\right)}^{\left(x+y+1\right)}}\kern0.5em {\displaystyle \begin{array}{c}C\left(y\le {y}_{\mathrm{min}}|x\right)=\sum \limits_{y=0}^{y\le {y}_{\mathrm{min}}}p\left(x|y\right)\\ {}D\left(y\ge {y}_{\mathrm{max}}|x\right)=\sum \limits_{y\ge {y}_{\mathrm{max}}}^{\infty }p\left(x|y\right)\end{array}} $$

### Prediction and analysis of miRNA target genes

Target genes of DEMs were identified using the miRanda [28], RNAhybrid [29] and TargetScan [30], and overlapping DEMs from all three programs provided the final results. The rules used for target prediction were based on those suggested previously by Allen et al.*..* [31] and Schwab et al [32], as follows: (1) No more than four mismatches between sRNA and target (G-U bases count as 0.5 mismatches); (2) No more than two adjacent mismatches in miRNA/target duplex; (3) No adjacent mismatches in positions 2–12 of the miRNA/target duplexes (5′ ends of miRNA); (4) No mismatches in positions 10–11 of miRNA/target duplex; (5) No more than 2.5 mismatches in positions 1–12 of the of miRNA/target duplexes (5′ ends of miRNAs); (6) Minimum free energy (MFE) of miRNA/target duplexes≥75% of MFEs of miRNAs bound to perfect complementary sequences.

Functions strongly associated with the predicted target genes of miRNAs were determined using Gene Ontology (GO; http://www.geneontology.org) [33] biological process categories, and Kyoto Encyclopedia of Genes and Genomes (KEGG) enrichment analysis was conducted using pathway database (http://www.genome.jp/kegg/pathway.html) [34] to statistically test DEMs.

### QRT-PCR and analysis of reference miRNAs

Total RNA was extracted as described above. For reverse-transcription of miRNAs, the PrimeScript RT Reagent Kit (Takara Bio, Dalian, China) were used. qRT-PCR was performed on a CFX-96 Real-time PCR System (Bio-Rad, CA, USA) in 25 μL reactions containing 12.5 μL SYBR Advantage Premix (2×) reagent (Takara Bio), 0.5 μL miRNA-specific forward primer (10 μM), 0.5 μL miScript universal primer (10 μM), and 2 μL PCR template (cDNA). Amplification was performed with an initial denaturation at 95 °C for 10s, followed by 40 cycles at 95 °C for 5 s and 60 °C for 20s, and a final cycle from 95 °C to 65 °C. All reactions were conducted in triplicate, which six biological replicates. The relative expression levels of DEMs were measured in terms of threshold cycle value (Ct) and were normalized to *U6 snRNA* using the eq. 2-ΔΔCt method [35]. Candidate reference miRNAs used for evaluation included *let-7a*, *miR-140-3p*, *miR-21*, *miR-15c*, *miR-26b*, *miR-92-5p*, and *miR-145a-5p* due to their low expression differences according to the Illumina deep sequencing results (data not shown). Additionally, *5 s rRNA*, *18 s rRNA* and *U6 snRNA* were also selected for evaluation as reference genes due to their broad use in the literature. QRT-PCR methods were the same as described above for DEMs, and the stability of reference miRNAs was tested using geNorm [36], NormFinder [37] and Bestkeeper [38] programs. All primers used in qPCR experiments (Additional file 1: Table S1) were synthesized by Sangon Biotech. (Shanghai, China). Data were analyzed statistically with SPSS 20 (IBM, Chicago, IL, USA) by t-test. Thresholds for statistical significance were set at *P* < 0.05 (significant) and *P* < 0.01 (highly significant).

## Results

### Identification of Koi carp miRNAs via sRNA sequencing

To identify miRNAs expressed in the skin of Koi carp, BS, RS, and WS sRNA libraries were determined and analyzed by Illumina deep sequencing. A Total of 20,502,959, 16,916,198, and 19,839,224 reads were obtained from the BS, RS, and WS libraries, respectively. After filtering out low-quality reads, insert-null, reads < 18 nt, and removing adaptor sequences, 19,649,441 (96.01%), 16,193,916 (95.91%), and 18,981,507 (95.87%) clean reads were retrieved for further analysis (Additional file 2: Table S2). In order to annotate the different classes of sRNAs, clean reads were compared with GenBank, Rfam, miRbase, exon and intron, and repeat-associated RNA databases. Representations of different types of RNA sequences, including rRNAs, tRNAs, snRNAs, snoRNAs, and scRNAs are shown in Table 1.

**Table 1:** Distribution of sequenced reads from raw data to cleaned sequences

Type	Black skin		Red skin		White skin	
	Total reads	%	Total reads	%	Total reads	%
Raw reads	20,502,959	100	16,916,198	100	19,839,224	100
tRNA	173,761	0.88	247,404	1.53	221,565	1.17
snoRNA	4455	0.023	4818	0.029	5169	0.027
snRNA	3143	0.016	4346	0.027	4199	0.022
rRNA	175,928	0.90	150,346	0.93	180,280	0.95
scRNA	6980	0.036	7887	0.048	7890	0.042
Intro_antisense	503,101	2.56	437,439	2.70	558,702	2.94
Exon_antisense	197,485	1.01	186,048	1.14	168,655	0.889
Other	18,584,128	94.58	15,155,625	93.59	17,835,047	93.96

**Fig. 2:**
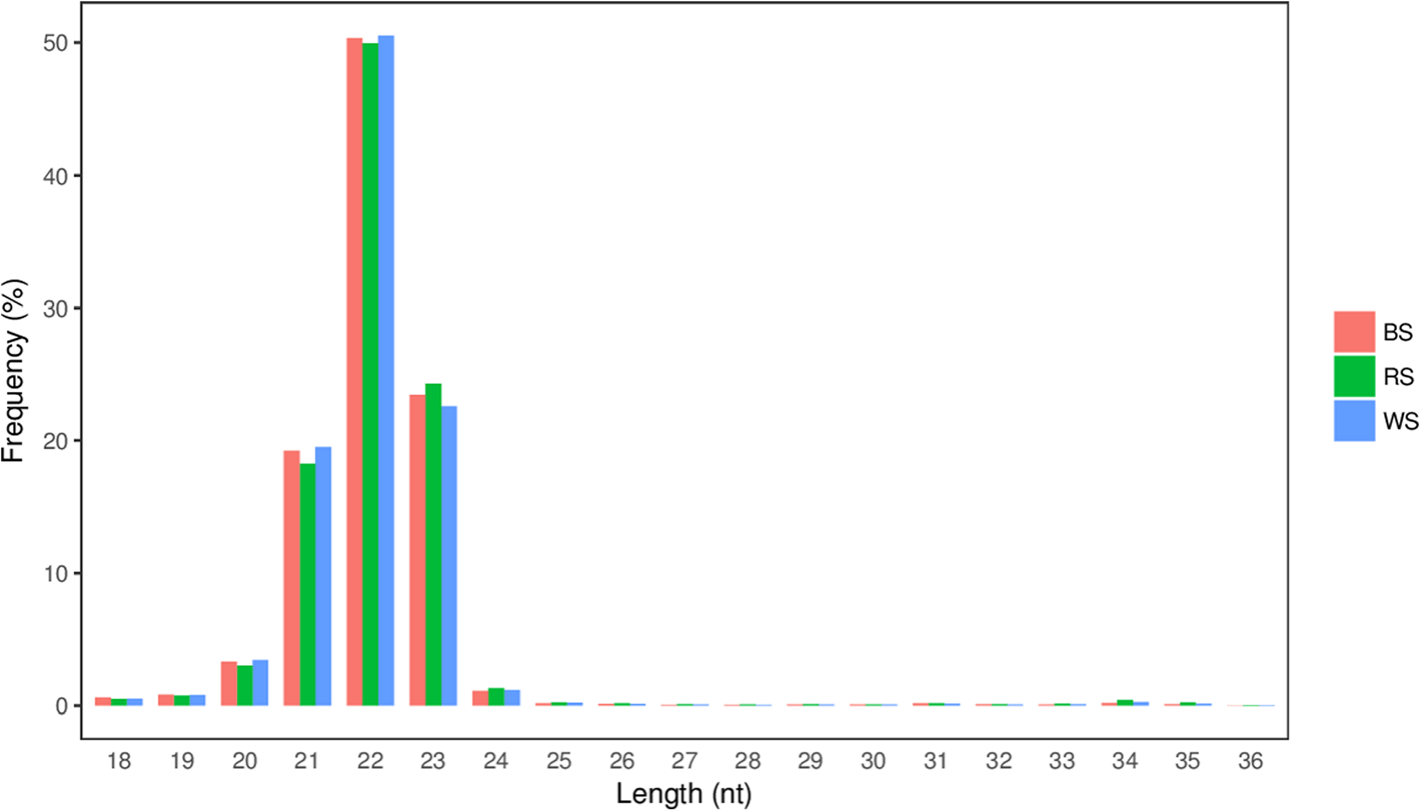
The length distribution of cleaned sequencing reads

The length distribution of sRNA reads is shown in Fig. 2. The results were similar among the three BS, RS, and WS groups, with 22- or 23- nt sRNAs accounting for 73.82, 74.20, and 73.10% of total sequences, respectively (Fig. 2).

### Characterization of miRNAs in different skin tissues

**Fig. 3:**
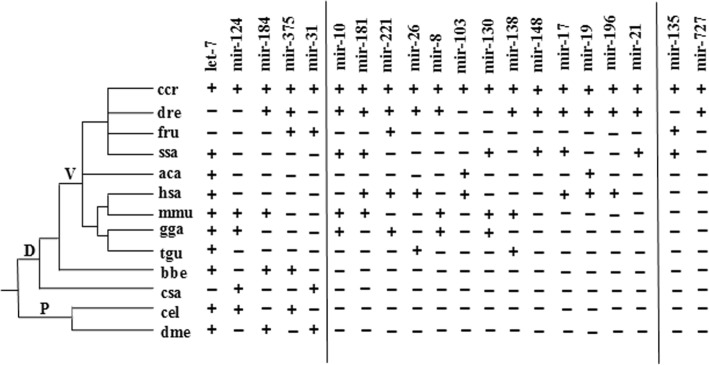
Phylogenetic tree of 20 conserved miRNA families in Koi carp. Note: The presence and absence of miRNAs is indicated by plus (+) and minus (−) symbols, respectively. Abbreviations: ccr, *Cyprinus carpio*; dre, *Danio rerio*; fru, *Takifugu rubripes*; ssa, *Salmo salar*; aca, *Anolis carolinensis*; hsa, *Homo sapiens*; mmu, *Mus musculus*; gga, *Gallus gallus*; tgu, *Taeniopygia guttata*; bbe, *Branchiostoma belcheri*; csa, *Ciona savignyi*; cel, *Caenorhabditis elegans*; dme, *Drosophila melanogaster*; P, Protostomia; D, Deutostomia; V, Vertebrata

To further identify conserved miRNAs and predict novel miRNAs in the three skin color tissues, all sRNA sequences were mapped to known miRNAs in miRBase 21.0. After alignment and additional sequence analysis, 330, 397, and 335 conserved miRNAs were identified from BS, RS, and WS libraries, respectively, that are highly conserved in other species (Additional file 3). These miRNAs exhibited a broad range of expression levels, ranging from 32,428,443 counts for the most abundant, to a single count. The results including known carp miRNAs in the miRBase database for common carp, including 137, 144, and 138 miRNAs for BS, RS, and WS groups, respectively, of which *miR-199-5p*, *miR-199-3p*, *let-7a*, *miR-21*, *miR-26a*, *miR-99*, *miR-100*, *miR-125b*, *miR-22a*, *miR-126-3p*, and *miR-146a* were the most abundant (Additional file 4). To obtain a clearer perspective of the most abundant known carp miRNAs, we compared those with the 10 highest read numbers (Table 2) and ~ 81 miRNA families were identified in each library. Several miRNA families were expressed at levels > 100,000 reads, such as *let-7*, *miR-199*, *miR-126*, *miR-27*, *miR-21*, etc. The degree of sequence conservation of miRNAs was assessed to investigate phylogenetic evolution, and 20 conserved families were clustered into three groups based on phylogenetic distribution (Fig. 3). Five miRNA families (*let-7*, *miR-124*, *miR-184*, *miR-375*, and *miR-31*) are shared in protostomes and deuterostomes, 13 families are presented only in vertebrate, and the other two families (*miR-135* and *miR-727*) have only been identified in fish.

**Table 2:** Ten most abundant known carp miRNAs identified in black, white, and red Koi carp skin samples

miRNA	Sequence	BS Count	RS Count	WS Count
ccr-miR-199-5p	CCCAGTGTTCAGACTACCTGTTC	4,100,063	3,122,326	3,587,226
ccr-miR-199-3p	ACAGTAGTCTGCACATTGGTT	1,730,490	1,389,612	1,653,539
ccr-let-7a	TGAGGTAGTAGGTTGTATAGTT	1,297,246	1,133,283	1,272,622
ccr-miR-21	TAGCTTATCAGACTGGTGTTGGC	1,309,364	1,138,255	1,255,244
ccr-miR-26a	TTCAAGTAATCCAGGATAGGCT	1,086,517	902,538	1,092,863
ccr-miR-99	AACCCGTAGATCCGATCTTGT	809,551	750,404	781,225
ccr-miR-100	AACCCGTAGATCCGAACTTGT	877,228	697,664	706,497
ccr-miR-125b	TCCCTGAGACCCTAACTTGTGA	742,182	506,300	590,501
ccr-miR-22a	AAGCTGCCAGCTGAAGAACTGT	529,973	402,752	535,663
ccr-miR-126-3p	CTCGTACCGTGAGTAATAATGC	485,109	394,630	476,118

We also found 1563 miRNAs not previously identified in Koi carp, comprising 340, 353, and 351 in BS, RS, and WS groups, respectively (Additional file 5). These were considered specific candidate miRNAs for further investigation. The seven most abundant miRNAs (*novel-miRn0378*, *novel-miRn0924*, *novel-miRn1323*, *novel-miRn0321*, *novel-miRn0902*, *novel-miRn0547*, and *novel-miRn1422*) all had > 300 reads (Table 3). We also investigated the most abundant novel miRNAs in each skin tissue, and *novel-miRn0022* and *novel-miRn0032* were dominantly expressed in white skin rather than black or red skin, with 1165 and 1108 reads, respectively (Table 4).

**Table 3:** Seven most abundant novel miRNAs identified in black, white, and red Koi carp skin samples

miRNA	Sequence	BS Count	RS Count	WS Count
novel-miRn0378	CAAGCTTGTTTCTATGGGTCTC	1720	1693	1940
novel-miRn0924	TAGATCAGTGAACTTGCCTTTA	2056	1388	1664
novel-miRn1323	TCAGTCACCGTTCACTTACATT	1414	1129	1356
novel-miRn0321	TGGAAACATTCTACACTCTCAGA	1242	931	1163
novel-miRn0902	CAAGCTCGATTCTGTGGGTCT	1027	746	792
novel-miRn0547	AATGCAAGAACACATCCTGAGT	832	662	805
novel-miRn1422	ATTATGAACATGATATTGAAT	415	370	424

**Table 4:** Ten most abundant novel miRNAs differentially expressed in black, white, and red skin samples

miRNA	Sequence	BS Count	RS Count	WS Count
novel-miRn0022	TAAAATGGACCATTGACACTCT	750	531	1165
novel-miRn0032	TCTGCAACACGAAACTGTCTTA	522	47	1108
novel-miRn0678	AAGTTCTGTGGTCCACTCTGGCT	1328	337	569
novel-miRn1387	ACTGATTTCCTCTGGTGCTTGGA	1099	779	577
novel-miRn1534	TCACGCTGCGGATCAGATGCTC	804	629	501
novel-miRn0032	TAAAGAGAACCGCCGCAAACGC	307	53	170
novel-miRn0824	AACGATCTTTAAACATTAATCT	266	77	510
novel-miRn0849	TTCAAACGGACCATTGACATTC	97	131	238
novel-miRn1070	GATCGTGATGAAACTTTAACC	376	138	581
novel-miRn1199	TTCAGTGAAGATAATCTGTCC	139	55	84

### Expression profiling of miRNAs in different skin patterns

**Fig. 4:**
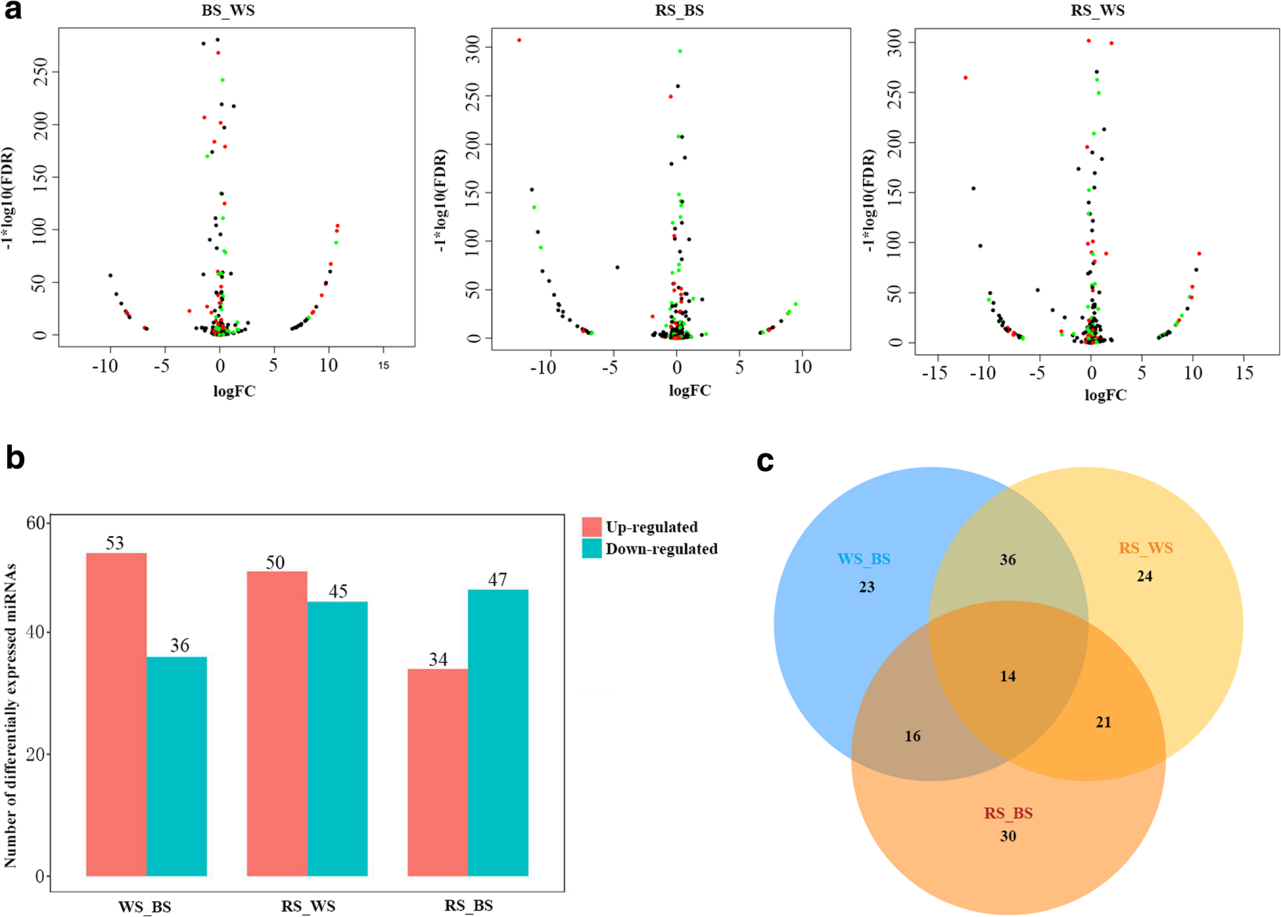
Differentially expressed miRNAs (DEMs) in white, red and black Koi carp skin. **a**, MA plot of differential miRNA expression levels among the three pairwise comparisons, black, red, and green dots represent non-significant, up-regulated, and down-regulated miRNAs, respectively; **b**, Number of DEMs among the three pairwise comparisons, red and blue indicate the number of up- and down-regulated miRNAs, respectively; **c**, Venn diagram comparing the expression distribution of miRNAs

**Fig. 5:**
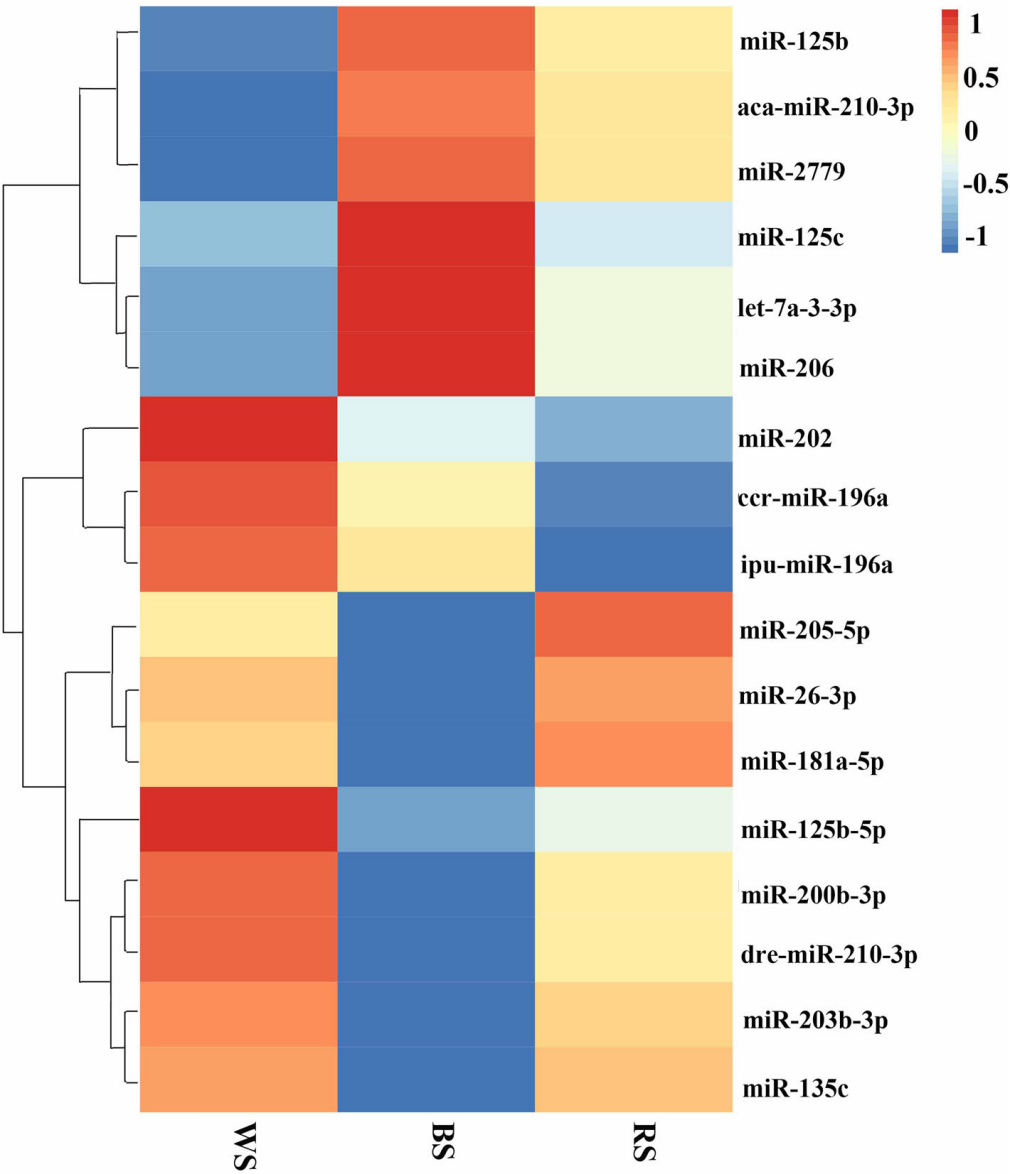
Heatmap of 17 known DEMs in WS, BS and RS groups. Note: The 17 rows and three columns correspond to each miRNA and skin tissue, respectively. The heatmap was drawn based on log10 normalized expression values for each miRNA in relation to expression in WS, BS, and RS skin groups

In the high-throughput sequencing analysis, many miRNAs exhibited dissimilar expression levels among the three skin color samples. Using the criteria |log_2_(Fold-change)| ≥ 1 and *q*-value ≤0.001, volcano plots of the three pairwise comparisons (BS vs. WS, RS vs. BS, and RS vs. WS) revealed the expression trends for each pair (Fig. 4a). We also constructed a histogram of DEMs in the three skin tissues (Fig. 4b). Compared with black skin, 53 miRNAs were up-regulated in white skin, including *miR-200b* and *miR-135c*, while 36 miRNAs were down-regulated. A total of 50 miRNAs were down-regulated in white skin compared with red skin, including *miR-217*, while 45 miRNAs were up-regulated. There were 47 miRNAs displaying greater abundance in black skin compared with red skin, including *miR-196a*, while 34 miRNAs were down-regulated (Additional file 6). The proportion of DEMs reflects their specific functions and related biological mechanisms in different skin tissues. A Venn diagram (Fig. 4c) was generated to visually compare the expression of miRNAs in Koi carp. Among the significant DEMs, 164 miRNAs (including 14 overlapping DEMs) were identified, and 30 miRNAs (including 17 conserve and 13 novel DEMs) were significant DEMs in WS and RS group compared with the BS group, suggesting that they likely play a key role in the color variation process (Fig. 4c). The heatmap of these 17 known DEMs (Fig. 5) indicates that eight miRNAs including *miR-203b-3p*, *miR-26-3p*, *miR-205-5p*, etc. were up-regulated in WS and RS groups compared with the BS group, while six miRNAs, including *aca-miR-210-3p*, *miR-125c*, *miR-206*, etc. were down-regulated in WS and RS groups compared with BS group. The remaining miRNAs (*ccr-miR-196a*, *miR-202*, and *ipu-miR-196a*) exhibited different expression trends (Fig. 5).

**Fig. 6:**
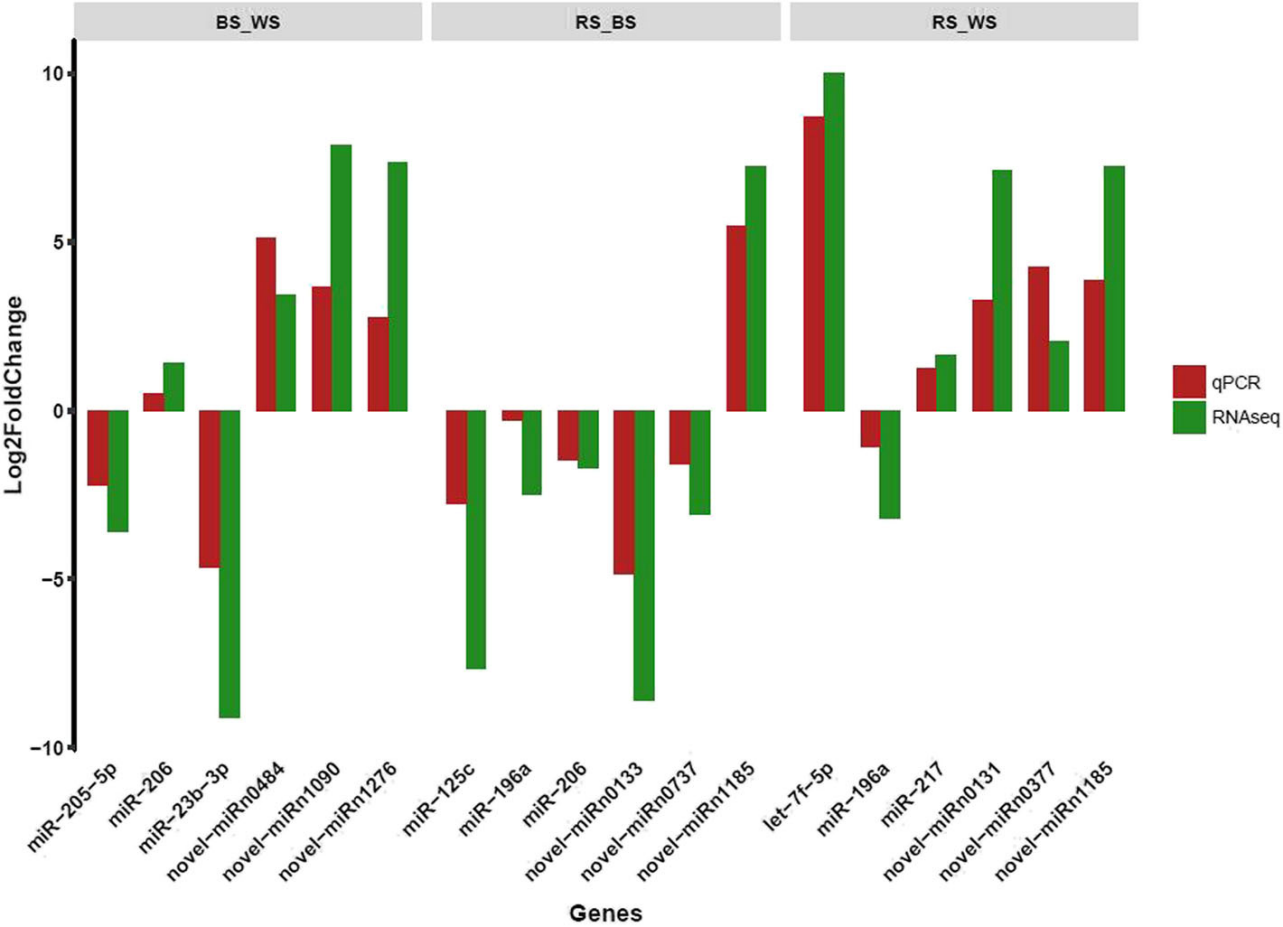
Comparison of fold change in expression between qRT-PCR and RNA-seq data

In order to validate the reliability of the Illumina sequencing results, expression in different skin tissues was examined by qRT-PCR for 18 miRNAs, including conserved and specific miRNAs, selected randomly from the dataset. The results showed that qRT-PCR expression patterns of all selected miRNAs were absolutely in agreement with the results of RNA-seq analysis, indicating that the RNA-seq data was reliable (Fig. 6). For example, based on the deep sequencing result, the expression level of *let-7f-5p* in RS was almost 10 times higher than in WS, compared with an 8-fold difference in the qRT-PCR results.

### Target genes prediction and signaling pathways analysis

Target genes were predicted based on the common carp (*C. carpio*) genome sequence (http://www.fishbrowser.org/database/Commoncarp_genome/) using a combination of TargetScan, RNAhybrid, and miRandato refine and improve the prediction results. A total of 2495 putative target genes were predicted, and some unique miRNAs, such as *miR-206, miR-125c, miR-217, miR-205-5p,* and *novel-miRn1185*, appear to target hundreds of genes. Moreover, our results indicate that some genes may be regulated by more than one miRNAs, such as cytoglobin-1-like (No: DN51764) that is targeted by nine miRNAs. Target genes were subjected to GO analysis for functional classification (Additional file 7). The result showed that many miRNAs were assigned to pigmentation-related terms such as melanocyte migration (GO: 0097324), retinal pigment epithelium development (GO: 0010669), and melanosome transport (GO: 0032402). These genes enriched in pigmentation-related processes are informative and worthy a further study.

**Fig. 7:**
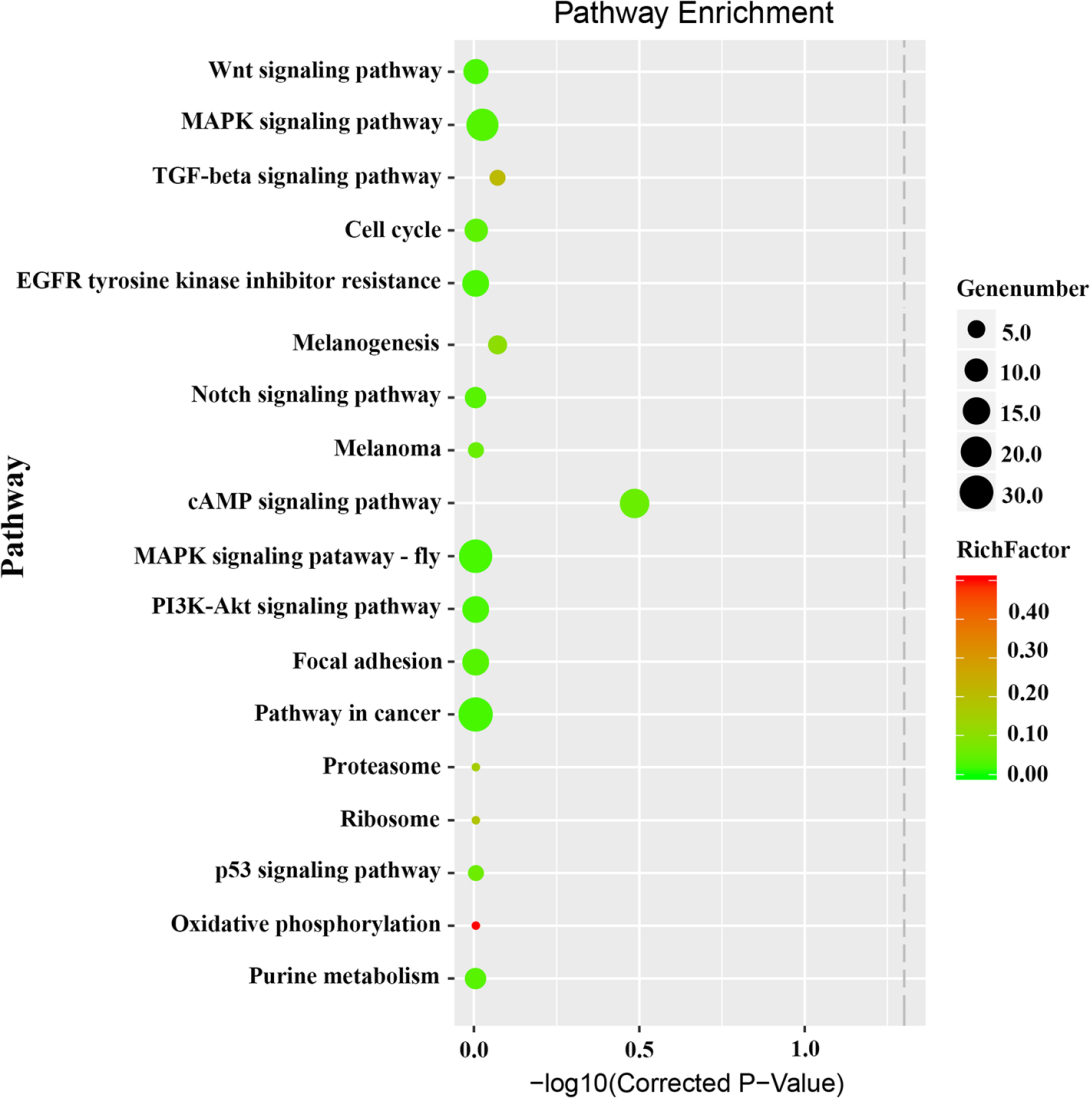
Predicted pigmentation-related pathways and target genes based on Kyoto Encyclopedia of Genes and Genomes (KEGG) pathway analysis. Gene number, number of target genes in each pathway; Rich factor; ratio of the number of target genes divided by the total number of genes in each pathway

**Fig. 8:**
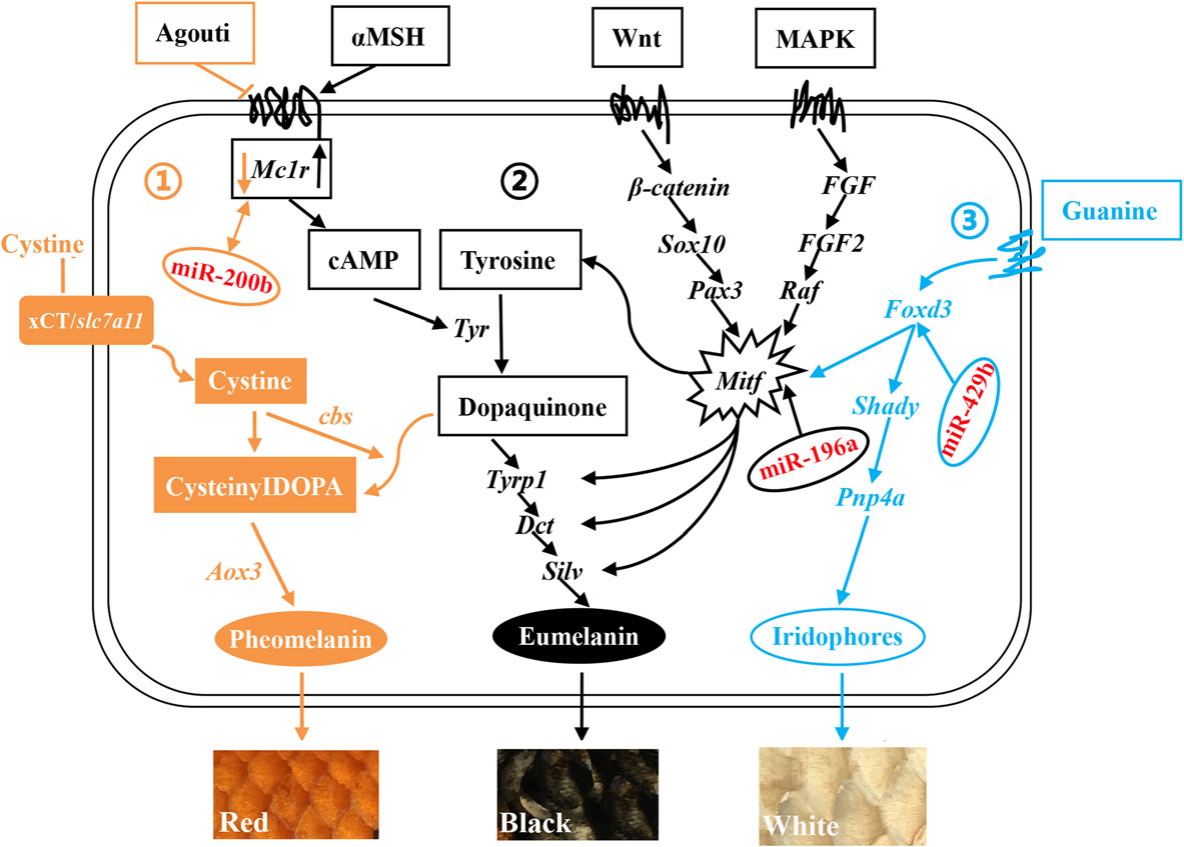
Schematic diagram of putative gene pathways related to the skin pigmentation process in Koi carp which drawn referred to reference literature [54, 55, 57]. (1) represents the pheomelanin biosynthesis pathway; (2) represents the eumelanin pathway. (3) represents the guanine pathway

KEGG pathway analysis was also performed to classify putative miRNA targets, and 275 annotated signaling pathways were found to be involved in a wide range of biological functions. For instance, at least 10 pathways involved in pigmentation-related biology were identified, including Wnt/β-catenin signaling, MAPK signaling, melanogenesis, oxidative phosphorylation, cell cycle, TGF-beta signaling, melanoma, cAMP signaling, epidermal growth factor receptor (EGFR) tyrosine kinase inhibitor resistance, and Notch signaling (Fig. 7). Herein, we mainly focused on melanogenesis, Wnt signaling, and MAPK signaling pathways, and putative gene pathways involved in different colored skin pigmentation process are shown in Fig. 8. Many genes in the melanogenesis pathway have been linked to skin color in Koi carp, including *Mc1r*, *Msh*, *Mitf*, kit oncogene (*KIT*), and *Tyr* [39]. We identified *Mc1r* as a target of *miR-200b*, *miR-206*, *miR-196a*, and *novel-miRn1185*. Meanwhile, *Msh* is a target of *miR-200a*, *miR-206*, *miR-142b-3p*, and *miR-26a-3p*. Wnt and MAPK signaling pathway are also involved in the pigmentation process in fish, and *Sox10*, *Fgf*, *Mitf*, and *Silv* are indispensable [40, 41]. These results showed that *miR-133c*, *miR-26a-3p*, *miR-196a*, and *miR-181a-5p* all target the *Mitf* gene. *Foxd3* is one of the earliest molecular makers in the Guanine pathway, and altering the expression level of this gene can control lineage choice between neural or glial and pigment cells, as demonstrated by repressing *Mitf* during the early phase of neural crest migration [42]. Herein, *Foxd3* was found to be targeted by *miR-429b, miR-26a-3p, miR-200b, miR-10b-5p*, and *miR-181a-5p*.

### Identification of optimal reference genes

**Fig. 9:**
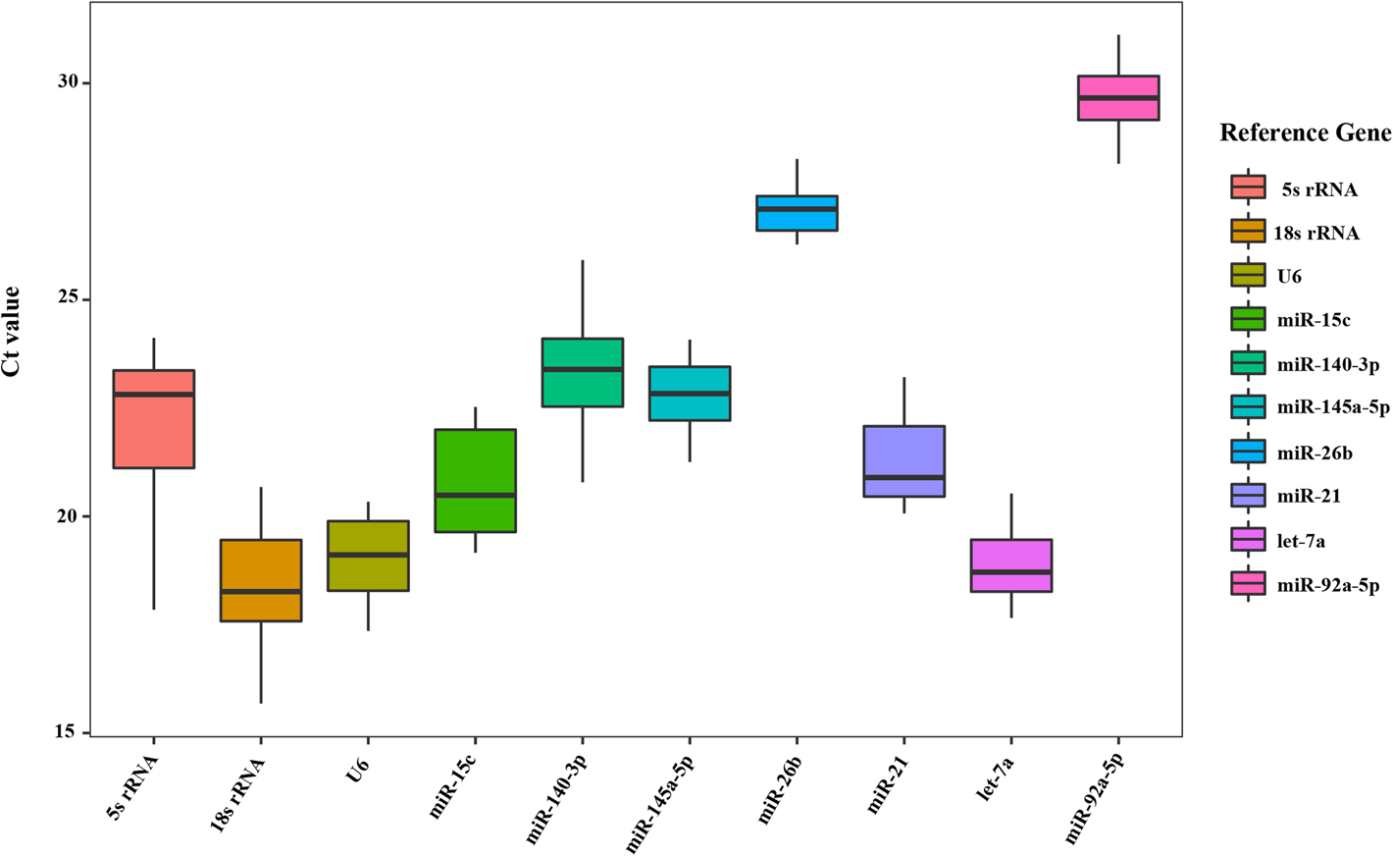
Box plot of the Ct values of the candidate reference genes. Note: Each box represents an internal candidate gene, with horizontal lines representing the median, the top of the box representing the third quartile, the bottom representing the first quartile, and lines crossing the box representing maximum and minimum values

As shown in Fig. 9, the Ct values of 10 genes (*let-7a*, *140-3p*, *21*, *15c*, *26b*, *92-5p*, *145a-5p*, *5 s rRNA*, *18 s rRNA*, and *U6*) ranged from 15.67 to 31.12 according to the qRT-PCR results, indicating that these 10 genes are expressed at normal levels in different tissues in Koi carp, and are therefore suitable as internal reference genes for screening purposes. *Let-7a* was the most highly expressed miRNA with the lowest Ct value, ranging from 17.65 to 19.89; and *18 s rRNA* had the lowest Ct value, while *miR-92a-5p* had the largest. The largest variation range was observed for *5 s rRNA* (6.28), while *miR-26b* (1.86) exhibited the least variation.

**Fig. 10:**
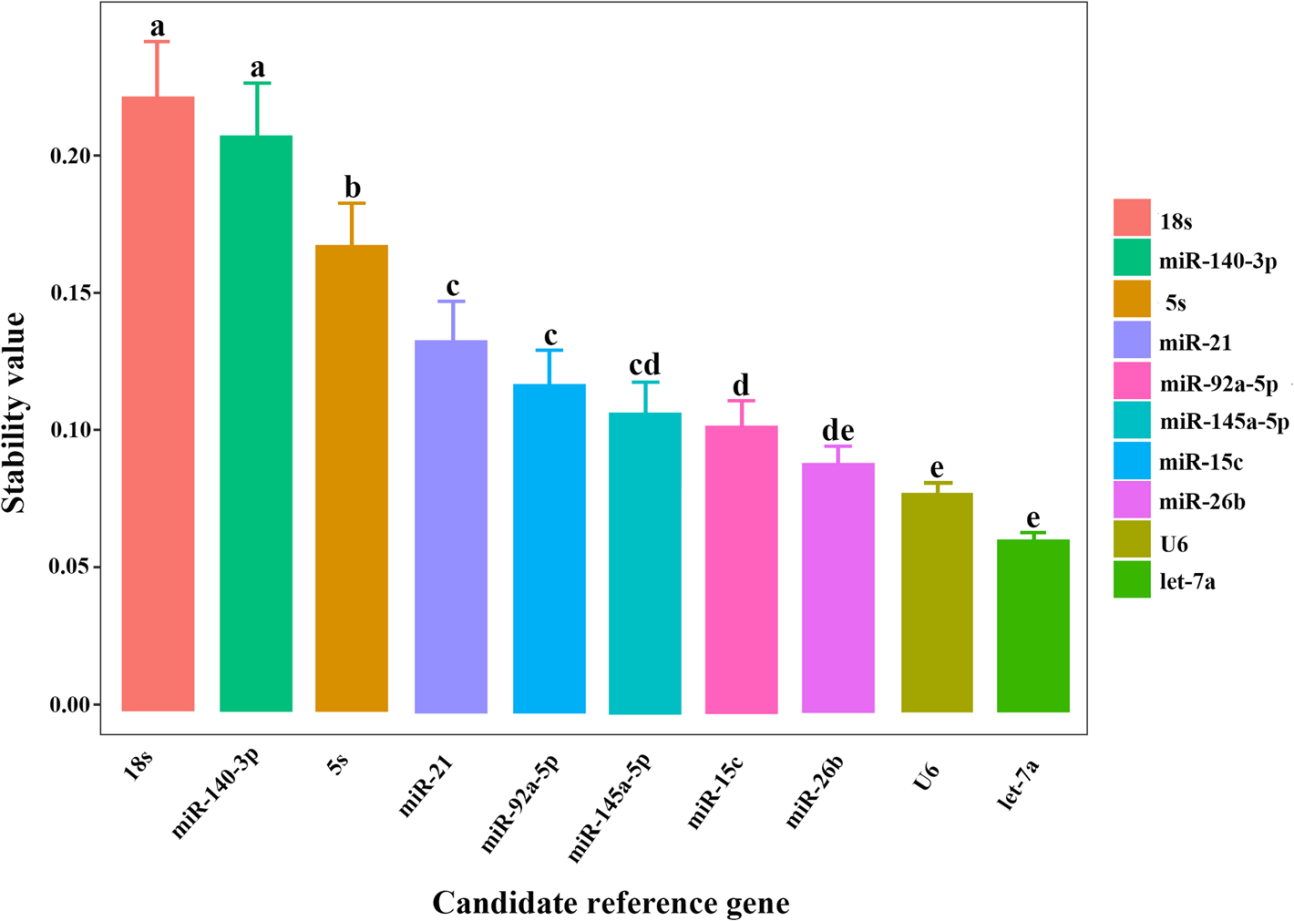
The stability of candidate reference genes analyzed by NormFinder. Values with a different superscript letter denote significant differences between genes (*P* < 0.05)

Analysis of miRNA expression stability (M) values using the geNorm algorithm was applied to each reference gene based on average pairwise variation between all candidate genes (M value is negatively correlated with miRNA stability; the lower the value, the higher the stability, and vice versa). The results showed that comparison of *let-7a* and *miR-26b* gave an M value of 0.661, which indicates high stability. The stability of the 10 reference genes in different tissues was ranked *let-7a* and *miR-26b* > *U6* > *miR-145a-5p* > *miR-21* > *miR-92a-5p* > *miR-15c* > *miR-140-3p* > *5 s rRNA* > *18 s rRNA* (Table 5). The NormFinder algorithm is similar to that used by geNorm, and the lower the expression of internal reference genes, the higher the stability. As shown in Fig. 10, the NormFinder reference gene stability in different Koi carp tissues was ordered *let-7a* > *U6* > *miR-26b* > *miR-15c* > *miR-145a-5p* > *miR-92a-5p* > *miR-21* > *5 s rRNA* > *miR-140-3p* > *18 s rRNA*, hence *let-7a* was the most stable gene. In BestKeeper analysis, based on average Ct values, the stability of internal reference genes should have a low standard deviation (*SD*) and coefficient of variation (*CV*), as well as a high Pearson correlation coefficient (*R*). The BestKeeper results showed that the best-performing internal reference gene was *let-7a*, followed by *miR-26b*, while the most unstable gene was *miR-140-3p*, followed by *18 s rRNA* (Table 6). Taking the results of these three analyses together, *let-7a* was the most stable single reference gene, *let-7a* and *miR-26b* was the most stable reference combination in different tissues, while *18 s rRNA* was the most unstable gene.

**Table 5:** Matrix of different reference gene combinations in different tissues

Gene name	*let-7a*	*miR-140-3p*	*miR-21*	*miR-15c*	*miR-26b*	*miR-92-5p*	*miR-145a-5p*	*5 s rRNA*	*U6*
*let-7a*	–	–	–	–	–	–	–	–	–
*miR-140-3p*	2.762	–	–	–	–	–	–	–	–
*miR-21*	1.577	2.311	–	–	–	–	–	–	–
*miR-15c*	2.332	1.298	2.562	–	–	–	–	–	–
*miR-26b*	0.661	2.864	1.824	2.218	–	–	–	–	–
*miR-92-5p*	1.811	1.783	2.042	1.726	1.756	–	–	–	–
*miR-145a-5p*	1.328	2.465	2.036	2.78	1.124	2.119	–	–	–
*5 s rRNA*	3.44	2.745	3.07	3.002	3.228	3.064	3.136	–	–
*U6*	0.985	3.228	1.756	3.11	0.867	2.198	1.033	3.176	–
*18 s rRNA*	3.541	3.186	3.426	3.464	3.398	3.345	3.422	2.033	3.348

Note: The left diagonal represents stability in different tissues, and — line represents no values

**Table 6:** Reference gene stability in different tissues based on BestKeeper analysis

Parameter	*let-7a*	*miR-140-3p*	*miR-21*	*miR-15c*	*miR-26b*	*miR-92-5p*	*miR-145a-5p*	*U6*	*5 s*	*18 s*
*R*	0.936	0.243	0.52	0.769	0.904	0.582	0.468	0.821	0.419	0.412
*SD*	0.866	0.83	1.426	1.377	0.824	1.124	1.59	1.12	1.396	2.114
*CV*	6.835	10.923	9.923	9.8	7.164	7.63	9.78	8.832	9.182	10.48

## Discussion

Body color and coloration patterns are prominent features related to intraspecific communication, interspecific interactions, photoprotection, photoreception, thermoregulation, and market value in many animals, including Koi carp. Thus, in the present work we explored the molecular mechanisms of pigmentation regulation and evolution to facilitate improving coloration and ornamental value [43]. Increasing evidence suggests that miRNAs play a vital role in skin pigmentation by controlling the expression of a vast number of genes [44, 45]. Herein, we generated miRNA transcriptome data from black, white, and red skin samples of Koi carp using a deep sequencing approach, and the findings provide a foundation for future skin pigmentation studies.

The BS, WS, RS miRNA libraries constructed from different colored skin tissue displayed a similar read length distribution of 22-23 nt miRNAs, equivalent in size to typical Dicer-derived products. The most abundant miRNAs were *let-7a*, *miR-21*, *miR-26a*, *miR-15c*, etc. with > 10,000 read each. These miRNAs are also highly expressed in other species; *let-7* family was first characterized in *Caenorhabditis elegans*, and plays a key role in regulating late developmental events by down-regulating *lin-41* and possibly other genes [46]; *miR-21* is an abundantly expressed miRNA in mammalian cells, and is evolutionarily conserved across a wide range of vertebrate species. Additionally, it is significantly up-regulated in gastric cancer, and it targets *15-PGDH*, suggesting the PGE2/PI3K/Akt/Wnt/β-catenin axis might be a novel pathway for gastric cancer treatment [47]. Thus, these miRNAs are considered to be housekeeping molecules involved in the maintenance of basic cellular activities.

To gain insight into the possible significance of skin miRNAs in Koi Carp, we determined expression profile of miRNAs in different colored (red, black, and white) skin tissues. We found that 30 miRNAs (17 conserved and 13 novel) were significant DEMs in WS and RS groups compared with the BS group, and 14 overlapping DEMs were shared by all three skin tissues, including *miR-196a*, *miR-125b*, *miR-202*, *miR-125c*, *miR-205-5p*, *miR-200b*, and etc. implying that these DEMs may therefore be involved in skin pigmentation. Knowledge of tissue- and cell-specific expression patterns of miRNAs could directly inform functional studies [48]. A previous study has revealed *miR-200b* and *miR-196a* as candidates that may be important for hair follicle (HF) development because their expression was reduced in the Dkk1 transgenic skin [49]. Meanwhile, *miR-125b* serves as a rheostat that controls stem cell proliferation, fate commitment, and differentiation [50]. Serum levels of *miR-205-5p* were determined by real-time PCR in 11 patients with metastatic melanoma and 16 patients without metastasis, and differences indicated that the miRNA network may be involved in the pathogenesis of melanoma metastasis [51]. Also, a significant correlation was observed between *miR-9-5p* and *miR-205-5p*, and between *miR-203a-3p* and *miR-205-5p*, suggesting that expression levels of these miRNA were not independently regulated in melanoma patients [51]. Herein, we found that *miR-196a*, *miR-202*, *miR-125b* and*miR-125c*, etc. were highly expressed in white and red skin compared with black skin, indicating specific functions in difference tissues. Thus, specific DEMs may regulate skin pigmentation in fish.

MiRNA target identification is important for predicting their functions. Although computational approaches have been widely used to predict targets, most methods suffer from a high false-positive rate [52]. Therefore, we used three software packages for analysis of target genes to refine and improve the results. We found that some miRNAs such as *miR-206*, *miR-125c*, *miR-217*, etc. targeted hundreds of genes, and some genes were regulated by more than one miRNA, indicating a complicated regulatory network between miRNAs and their targets gene. Skin pigmentation in fish is a complex process which involves numerous physiological, cellular and genetic factors [53]. Previous studies found that signaling pathway such as the Wnt, MAPK, cAMP, melanogenesis, Notch, and Fgf pathways strongly influence skin pigmentation process [41]. Herein, we constructed the putative gene pathways with some miRNAs involved in red, black, and white color skins pigmentation process. The molecular mechanism of melanin biosynthesis was extensively studied due to its biomedical significance, in mammals and birds, the ratio of eumelanin and pheomelanin largely determines an animal’s overall color: darker (black to brown) phenotypes result from the increased deposition of eumelanin, while lighter (red to yellow) phenotypes result from the increased deposition of pheomelanin [54]. Xu (2014) also elucidated the pigmentation mechanisms of Hebao and Songpu carp, in the melanogenesis pathway, tyrosine is oxidized to form dopaquinone, and then intracellularly catalyzed to become eumelanin; However, cystine and dopaquinone can switch off the eumelanin synthesis pathway and promote the synthesis of pheomelanin; And *slc7a11* encodes the transmembrane cystine/glutamate exchanger (xCT), which transports cystine into melanocytes to synthesize pheomelanin [55]. Both *miR-200* and *miR-205* are highly expressed in normal hair follicles compared epithelia tumors in human [56]. The *Mc1r* gene is a key factor involved in the production of pheomelanin and eumelanin in melanocytes, and we found that it is targeted by *miR-200a*, *miR-200b*, and *miR-206*, and *miR-206* presented up-regulated in black skins compared with red and white skins suggested that it played important roles in pigmentation in Koi carp. Wnt and MAPK are implicated in numerous development and physiological process. Sun reported that both Wnt and MAPK signaling pathways are highly likely to be involved in melanin biosynthesis in common carp [57]. *Mitf* is a member of the Myc-related family of basic helix-loop-helix leucine zipper (bHLH-Zip) transcription factors, and *Mitf* was found in cells related to the retinal pigment epithelium (RPE), as well as cells behind the optic cup that were probably derived from neural crest tissue and could develop into iridophore pigment cells [58]. Mutations of the *Mitf* gene causes a variety of phenotypes, most notably in pigmented cells [59]. *Foxd3* can affect the lineage between neural or glial and pigment cells by repressing *Mitf* during the early phase of neural crest migration [42]. Kevin et al.*.*. reported a model which melanophores and iridophores descend from a common precursor whose fate is regulated by the interplay transcription factors regulating *Mitf* and *Foxd3*, and loss of *Mitfa* resulted in supernumerary ectopic iridophores, while loss of *Foxd3*, a *Mitfa* repressor, resulted in fewer iridophores [60]. Double mutants displayed restoration of iridophores, suggesting that one of *Foxd3* is to suppress *Mitfa* to promote iridophore development [60]. In our present study, we also observed *miR-429b*, *miR-26a-3p*, and *miR-181a-5p* can target the *Foxd3* gene, and *miR-133c*, *miR-196a* act on the *Mitf* gene. Therefore, we have carried out a partial analysis of the regulatory network of the three color skins, while the specific mechanisms and functional need to be deeply excavated.

A number of reference genes for mRNA qRT-PCR analysis have been established, but miRNA qRT-PCR studies are fewer in number. Thus, we attempted to select suitable reference genes for normalization to eliminate or decrease non-biological variation between test samples. The two most commonly used reference genes for normalization in miRNA analyses are *U6* and *18S rRNA*, but there has been some debate as to the validity since they are expressed at levels greater than target genes [61, 62]. Xu et al (2014). found that *miR-101a* was the most stable miRNA when all tissue types were considered separately [63]. In *Siniperca chuatsi*, all six miRNAs (miR-101a, miR-146a, miR-22a, miR-23a, miR-26a and let-7a) exhibited better expression consistency than U6 in most of the conditions examined, and the best combination of reference genes was *miR-22a* and *miR-23a* [64]. Herein, we systematically evaluated the reliability of seven miRNA genes across 15 tissues. Our results suggest that *let-7a* was the most stable single reference gene, and the best combination of reference genes was *let-7a* and *miR-26b*, followed by *U6*, while *18 s rRNA* was the most unstable gene.

## Conclusions

Our findings provide fundamental information on the expression of conserved and potential novel miRNAs in Koi carp skin, and identified DEMs and their respective target genes that may be related to skin color. We identified 137, 144, and 138 miRNAs for BS, RS, and WS groups which already present in the *C.carpio* miRBase database, and *miR-203b-3p*, *miR-125c*, *ccr-miR-196a*, *miR-202*, and etc. were presented differences in expression in the three different skin color tissue samples. The best combination of reference genes was *let-7a* and *miR-26b*. Validation of the functional role of DEMs and reference genes will provide a better understanding of miRNA-mediated regulatory mechanisms influencing skin color in fish.

## Additional files

Additional file 1: **Table S1.** Primers for skin DEMs and reference miRNAs in Koi carp. (DOCX 14 kb)

Additional file 2: **Table S2.** Read statistics of the obtained small RNAs. (DOCX 13 kb)

Additional file 3: Conserved miRNAs identified in black, white, and red skin samples from Koi carp. (XLSX 91 kb)

Additional file 4: Known carp miRNAs identified in black, white, and red skin samples from Koi carp. (XLSX 45 kb)

Additional file 5: Novel miRNAs identified in black, white, and red skin samples from Koi carp. (XLSX 228 kb)

Additional file 6: Differentially expressed miRNAs (DEMs) identified in black, white, and red skin samples from Koi carp. (XLSX 40 kb)

Additional file 7: Predicted target genes of DEMs and target genes associated with significantly enriched Gene Ontology (GO) terms in Koi carp. (XLSX 208 kb)

## Abbreviations

Akt: Protein kinase B
cAMP: Cyclic Adenosine Monophosphate
cDNA: Complementary DNA
CV: Coefficient of variation
DEMs: Differentially expressed miRNAs
Dkk1: Dickkopf-related protein 1
EGFR: Epidermal growth factor receptor
FFRC: Freshwater Fisheries Research Center
Fgf: Fibroblast growth factor
Foxd3: Forkhead box D3
GAPDH: Glycer-aldehyde-3-phosphate dehydrogenase
GO: Gene ontology
HF: Hair follicles
KEGG: Kyoto Encyclopedia of Genes and Genomes
Kit: Kit oncogene
MAPK: Mitogen-activated protein kinase
Mc1r: Melanocortin receptor 1
MFE: Minimum free-energy
Mitf: Microphthalmia-associated transcription factor
mRNA: Messenger RNA
Msh: Melanocyte-stimulating hormone
NCBI: National Center for Biotechnology Information
Notch: Notch signaling pathway
Nt: Nucleotides
PGDH: Phosphoglycerate dehydrogenase
PGE2: Prostaglandin E2
PI3K: Phosphoinositide 3-kinase
qRT-PCR: Real-time quantitative reverse transcription PCR
R: Pearson correlation coefficient
RISC: RNA-inducing silencing complex
RPE: Retinal pigment epithelium
SD: Standard deviation
Silv: Silver locus protein homolog
Sox: SRY-related HMG-box
TGF-beta: Transforming growth factor beta
TPM: Transcripts per million
Tyr: Tyrosinase
Tyrp1: Tyrosine related protein-1
Wnt: Wingless and INT-1
