# Supplementary material for: Identification and characterization of skin color microRNAs in Koi carp (Cyprinus carpio L.) by Illumina sequencing
Source: BMC Genomics. 2018 Oct 29;19:779. doi: 10.1186/s12864-018-5189-5 (PMC6206873; doi:10.1186/s12864-018-5189-5)
Supplement: Supplementary file 1 — Table S1. Primers for skin DEMs and reference miRNAs in Koi carp. (DOCX 14 kb) [file 12864_2018_5189_MOESM1_ESM.docx]

**Additional file 1: Table S1** Primers for skin DEMs and reference miRNAs in Koi carp

| **Name** | **Sequence** |
| --- | --- |
| miR-196a | GCGCGCTAGGTAGTTTCATGTT |
| miR-205-5p | GCGTCCTTCATTCCACCG |
| miR-217 | GCGCTACTGCATCAGGAACTG |
| let-7f-5p | GCGCGCTGAGGTAGTAGATTGT |
| miR-206 | GCGCGTGGAATGTAAGGAAGT |
| miR-23b-3p | GCATCACATTGCCAGGGATT |
| miR-125c | GCGTCCCTGAGACCCTAACT |
| novel-miRn0484 | GCGCAGTGAAAGACTTTCCC |
| novel-miRn1090 | CGCGAAGTCAATGGGAACC |
| novel-miRn1276 | GCGTCCTATTTCTGCCCTG |
| novel-miRn0133 | GCGCGCTTCCTATGCATATAC |
| novel-miRn0737 | ATTATGAATGTTGATATTGCGT |
| novel-miRn1185 | ATATCCCGGACGAGCCCCCA |
| novel-miRn0131 | GCTAATACTGTCTGGTAATGCC |
| novel-miRn0377 | ATTATGAACATCGATCTTGCGT |
| let-7a | GCGCGTGAGGTAGTAGGTTGT |
| miR-140-3p | GCGCTACCACAGGGTAGAAC |
| miR-21 | GCGCGTAGCTTATCAGACTGGT |
| miR-15c | GCAAGCAGCGCGTCATG |
| miR-26b | GCGCTTCAAGTAATCCAGGA |
| miR-92a-5p | GCAGGTTGGGATTGGTAGC |
| miR-145a-5p | GCGTCCAGTTTTCCCAGGA |
| 5S-F | CTTACGGCCATACCACCCTG |
| 5S-R | CTGCTGCAAAGTGTGGGCTA |
| 18S-F | GCTCGTAGTTGGATCTCGGG |
| 18S-R | GCCGGAGTCTCGTTCGTTAT |
| U6-F | CTCGCTTCGGCAGCACA |
| U6-R | AACGCTTCACGAATTTGCGT |
