# Supplementary material for: Identification and characterization of skin color microRNAs in Koi carp (Cyprinus carpio L.) by Illumina sequencing
Source: BMC Genomics. 2018 Oct 29;19:779. doi: 10.1186/s12864-018-5189-5 (PMC6206873; doi:10.1186/s12864-018-5189-5)
Supplement: Supplementary file 2 — Table S2. Read statistics of the obtained small RNAs. (DOCX 13 kb) [file 12864_2018_5189_MOESM2_ESM.docx]

**Additional file 2, Table S2**: Read statistics of the obtained small RNAs

| **Samples** | **Total reads** | **Clean reads** |
| --- | --- | --- |
| Black skin | 20,502,959 | 19,649,441 |
| Red skin | 16,916,198 | 16,193,916 |
| White skin | 19,839,224 | 18,981,507 |
